# Non-Alcoholic Wernicke–Korsakoff Syndrome After a Whipple Procedure: Delayed Recognition, Neuropsychological Findings, and Rehabilitation Follow-Up: A Case Report

**DOI:** 10.3390/reports9030290

**Published:** 2026-09-01

**Authors:** Ülkü Figen Demir, Fatmanur Karakuş Dilbaz, Nur Banu Memur, Cahit Keskinkılıç, Aylin Hasanefendioğlu Bayrak, Muharrem Battal

**Affiliations:** 1 Department of Neurology, Faculty of Medicine, Istanbul Yeni Yüzyıl University, 34010 Istanbul, Türkiye; 2 Department of Internal Medicine, Faculty of Medicine, Istanbul Medeniyet University, 34700 Istanbul, Türkiye; 3 Department of Neuropsychology, Istinye University, 34250 Istanbul, Türkiye; 4 Department of Radiology, Faculty of Medicine, Istinye University, 34250 Istanbul, Türkiye; 5 Independent Practice, General Surgery, 34000 Istanbul, Türkiye

**Keywords:** Wernicke encephalopathy, Korsakoff syndrome, Whipple procedure, cognitive rehabilitation, case report

## Abstract

**Background and Clinical Significance**: Wernicke encephalopathy (WE) is a potentially reversible neurological emergency caused by thiamine deficiency. Although classically associated with alcohol use, it may also occur after gastrointestinal surgery and prolonged nutritional compromise, where diagnosis can be obscured by postoperative delirium or metabolic encephalopathy. **Case Presentation**: A 58-year-old woman with no history of alcohol use underwent a Whipple procedure for pancreatic head adenocarcinoma. Her postoperative course included an anastomotic leak, prolonged open-abdomen management, severely restricted oral intake, and long-term total parenteral nutrition. Approximately 1.5 months after surgery, she developed fluctuating confusion, visual hallucinations, marked recent-memory impairment, diplopia, nystagmus, and gait ataxia, initially interpreted as postoperative delirium/metabolic encephalopathy. Approximately two years later, persistent anterograde amnesia, cerebellar and oculomotor findings, and neuropsychological deficits in attention, memory, and executive functions led to clinical suspicion of previous WE. Re-evaluation of the acute MRI showed bilateral medial thalamic and periaqueductal FLAIR hyperintensities that were absent on follow-up imaging, supporting the retrospective diagnosis; the persistent amnestic syndrome was compatible with Korsakoff syndrome. B-complex vitamin supplementation was initiated, followed by 10 weekly 60 min cognitive rehabilitation sessions and a family-supported home program. Family members reported improved retention of recent information and greater daily independence, but formal post-treatment neuropsychological reassessment could not be completed. **Conclusions**: This case highlights the difficulty of recognizing non-alcoholic WE during a complicated postoperative course. The observed functional changes cannot be attributed specifically to rehabilitation because vitamin supplementation, nutritional recovery, spontaneous recovery, and rehabilitation occurred within the same follow-up period.

## 1. Introduction and Clinical Significance

Wernicke encephalopathy (WE) is an acute neurological disorder caused by thiamine (vitamin B1) deficiency and represents a potentially reversible medical emergency when recognized and treated promptly. Although traditionally associated with chronic alcohol use, WE also occurs in a wide range of non-alcohol-related conditions, including prolonged malnutrition, persistent vomiting, malignancy, malabsorption, gastrointestinal surgery, and prolonged parenteral nutrition [1,2,3]. In alcohol-associated WE, thiamine deficiency may result from a combination of inadequate nutritional intake, impaired gastrointestinal absorption, reduced hepatic storage, and altered thiamine utilization. In non-alcoholic WE, by contrast, deficiency most often develops in the setting of markedly reduced intake, impaired absorption, increased metabolic requirements, or prolonged nutritional compromise [1,2].

The underlying biochemical consequences of thiamine deficiency and the major neurological manifestations are broadly similar in alcoholic and non-alcoholic WE. Both forms may present with altered mental status, oculomotor abnormalities, and gait or truncal ataxia; however, the complete classic triad is present in only a minority of patients [2,4]. This diagnostic limitation is particularly important in non-alcoholic and postoperative patients, in whom confusion, behavioral changes, or cognitive impairment may initially be attributed to delirium, metabolic encephalopathy, infection, medication effects, or the underlying systemic illness [1,5]. Neuroimaging findings also overlap substantially, although atypical MRI involvement has been reported more frequently in non-alcoholic WE [5,6]. Treatment principles are similar regardless of etiology and are based on the prompt administration of parenteral thiamine together with correction of the underlying nutritional disorder; treatment should not be delayed while awaiting biochemical confirmation when clinical suspicion is high [7].

When WE is unrecognized or inadequately treated, persistent memory impairment and executive dysfunction may evolve into Korsakoff syndrome, characterized predominantly by severe anterograde amnesia with variable retrograde memory impairment and other cognitive deficits [8]. Gastrointestinal procedures, including pancreaticoduodenectomy (Whipple procedure), may create a particularly vulnerable setting because postoperative complications can result in prolonged restriction of oral intake, malabsorption, catabolic stress, and dependence on parenteral nutrition [9,10]. In such patients, the manifestations of WE may be obscured by a complex postoperative course and the diagnosis may therefore be substantially delayed.

The present case describes a woman with no history of alcohol use who developed an acute clinical syndrome compatible with WE after a complicated Whipple procedure; however, the diagnosis was established retrospectively approximately two years later, by which time a persistent amnestic syndrome compatible with Korsakoff syndrome was evident. The case provides a longitudinal description integrating the postoperative nutritional history, retrospective neuroimaging findings, detailed neuropsychological characterization, vitamin supplementation, and subsequent cognitive rehabilitation follow-up. Particular emphasis is placed on the diagnostic challenges of delayed recognition and on the distinction between objectively documented baseline cognitive impairment and subsequent informant-reported functional change.

## 2. Case Presentation

A 58-year-old woman underwent a Whipple procedure after biopsy of a pancreatic head mass demonstrated adenocarcinoma. Her medical history was notable for hypertension treated with nebivolol. She had never consumed alcohol.

The first two postoperative weeks were uneventful. Subsequently, fever prompted further evaluation, and an anastomotic leak was identified. Because of the complicated postoperative course, the patient was managed for approximately four months with an open abdomen and an intra-abdominal vacuum system. During this period, oral intake was severely restricted; according to the available clinical history, much of the limited fluid and enteral intake was removed through the vacuum system, and nutritional support was primarily provided by total parenteral nutrition (TPN). The available discharge documentation confirmed the use of prolonged TPN; however, detailed records of the TPN formulation were not accessible. Therefore, whether the parenteral regimen contained multivitamin or thiamine supplementation, and at what dose, could not be retrospectively verified.

Approximately 1.5 months after surgery, the patient developed fluctuating confusion, visual hallucinations, and marked impairment in recent memory. Her family reported that she sometimes perceived her children as being much younger and described seeing people who were not present. These symptoms fluctuated for approximately 10 days, after which difficulty retaining newly acquired information became particularly prominent. She frequently could not recall events that had occurred even approximately one hour earlier, whereas memories of childhood and remote autobiographical events remained relatively preserved.

During the same period, she developed dizziness, diplopia, jerky eye movements, and marked gait unsteadiness, eventually becoming unable to walk without assistance. Neurological and psychiatric assessments and cranial imaging were performed during hospitalization. In the context of the severe postoperative complications, prolonged hospitalization, and systemic illness, the neuropsychiatric presentation was initially interpreted as postoperative delirium/metabolic encephalopathy, and symptomatic treatment was recommended.

Approximately 5.5 months after surgery, the patient’s general medical condition improved sufficiently for discharge. Her family subsequently paid particular attention to improving her oral nutritional intake with a protein- and vitamin-rich diet. No additional medically prescribed nutritional supplementation was reported during this post-discharge period before her later neurological assessment. The visual misperceptions gradually diminished, and dizziness and diplopia substantially improved within approximately one month. However, marked difficulty retaining recent information and persistent balance impairment remained.

The clinical course and major diagnostic and therapeutic milestones are summarized in Table 1.

Approximately two years after surgery, the patient was evaluated at our neurology clinic because of persistent memory and balance problems. She was alert, oriented, and cooperative, although her reaction time was mildly prolonged. Speech showed cerebellar dysarthria, while comprehension was preserved. On brief bedside memory testing, immediate registration of three words was preserved, whereas after a delay of approximately 3–5 min she recalled only one of the three words. Cueing and recognition did not materially improve retrieval. Remote autobiographical memory, particularly for childhood and life events preceding surgery, was relatively preserved, whereas there was a marked memory gap for events occurring during the prolonged hospitalization. The clinical pattern was considered consistent with prominent anterograde amnesia. Neurological examination demonstrated bilateral gaze-evoked nystagmus without a major limitation of ocular movements. Muscle strength was normal in all extremities, with mildly decreased tone and bilaterally hypoactive deep tendon reflexes. An anteroposterior head tremor and intention tremor of both upper extremities were present. The patient stood with a wide base and could walk without physical assistance but showed prominent side-to-side instability.

**Figure 1:**
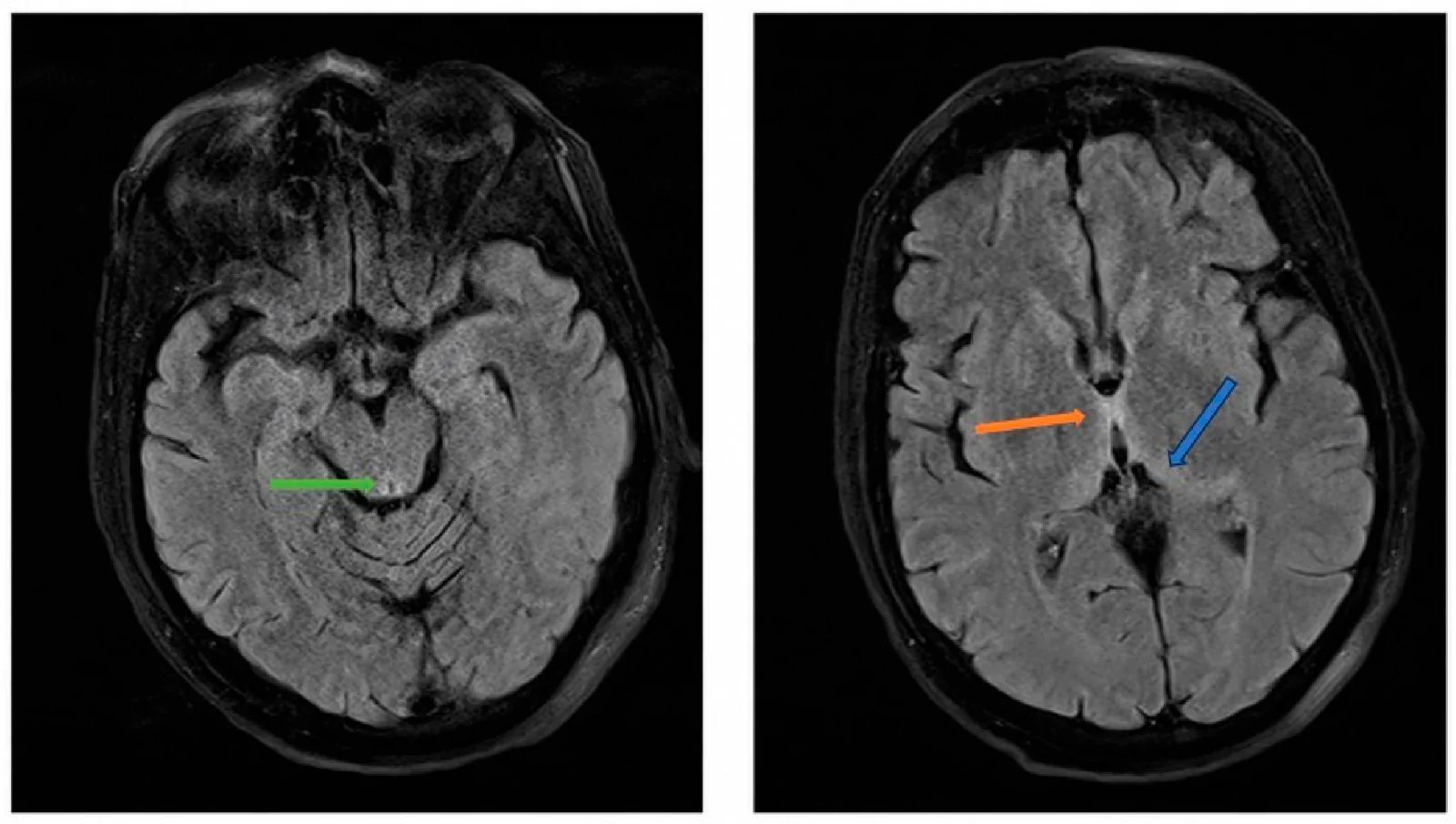
Early axial FLAIR MRI images obtained during hospitalization. Bilateral medial thalamic and periaqueductal FLAIR hyperintensities are shown. The green arrow indicates the periaqueductal FLAIR hyperintensity, whereas the orange and blue arrows indicate the bilateral medial thalamic FLAIR hyperintensities. In the subsequent clinical context, these findings were considered compatible with Wernicke encephalopathy.

Laboratory data sufficient to assess thiamine status during the acute postoperative phase were not available; however, in our evaluation, the combination of the patient’s clinical history, neurological examination findings, and neuropsychological test results raised suspicion of a previous episode of Wernicke encephalopathy during the postoperative period. The cranial MRI obtained during the acute postoperative hospitalization, which had originally not been reported as showing findings suggestive of WE, was therefore retrospectively re-examined. Bilateral medial thalamic and periaqueductal FLAIR hyperintensities were identified (Figure 1).

**Figure 2:**
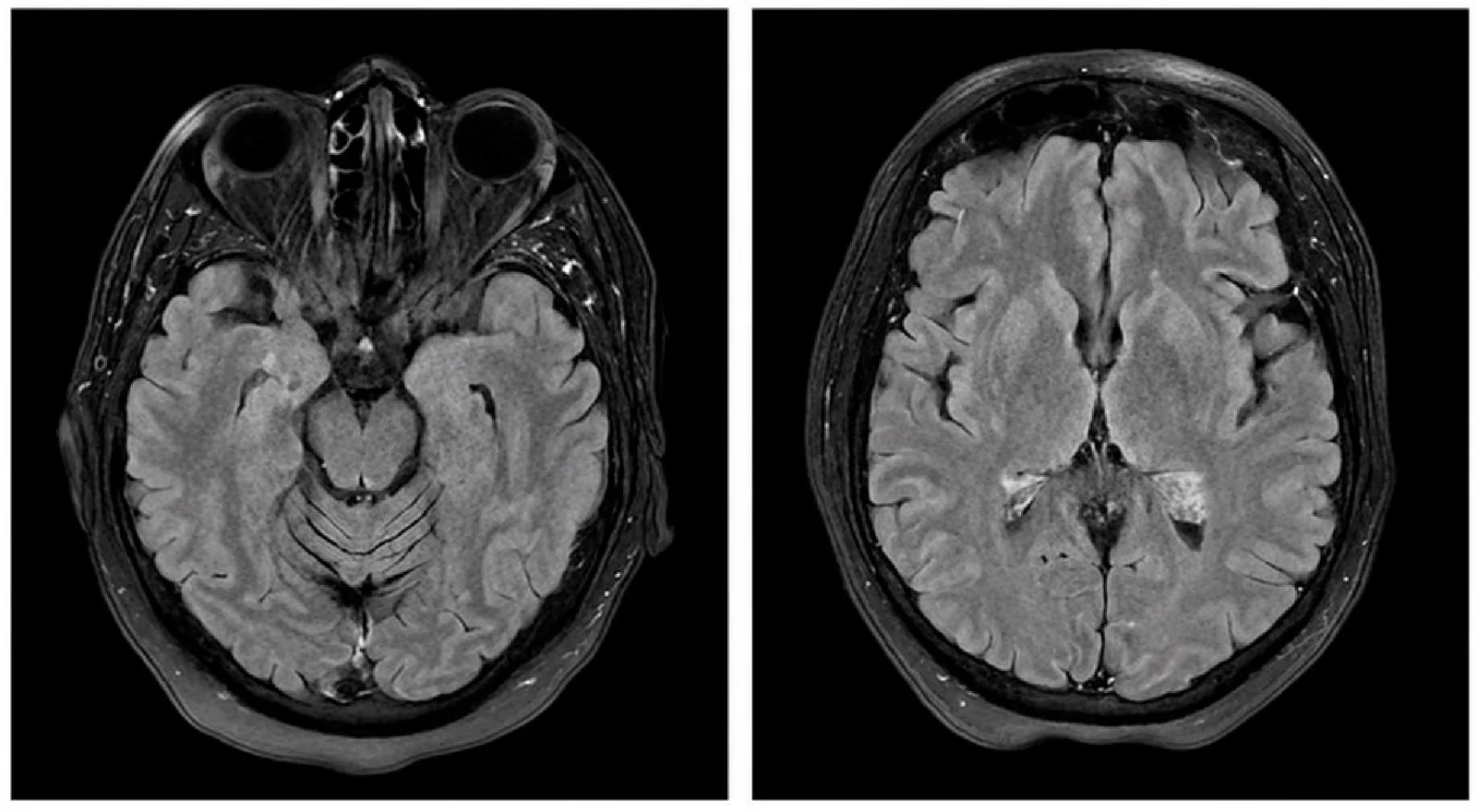
Follow-up axial FLAIR MRI images obtained approximately two years later. The previously observed periaqueductal and bilateral medial thalamic hyperintensities are no longer evident.

The acute-phase images were reviewed with the radiologist involved in the present report, who agreed that the anatomical distribution was compatible with WE. A follow-up cranial MRI obtained approximately two years later no longer demonstrated these abnormalities (Figure 2). To further assess the imaging findings, the acute-phase and follow-up MRI examinations were additionally reviewed by an independent radiologist who was not involved in the study and was initially blinded to the patient’s clinical history and diagnostic hypothesis. On this initial imaging-only assessment, the radiologist considered the abnormalities on the acute-phase MRI to be pathological and confirmed that they were no longer present on the follow-up MRI. After the clinical history and the diagnostic suspicion of Wernicke encephalopathy were subsequently disclosed, the radiologist agreed that the distribution of the abnormalities was compatible with WE. Taken together with the prolonged nutritional vulnerability, acute mental-status changes, oculomotor abnormalities, gait ataxia, and persistent amnesia, the acute neurological syndrome was considered retrospectively compatible with WE, whereas the later persistent amnestic and executive syndrome was considered compatible with residual Korsakoff syndrome.

A formal neuropsychological assessment was subsequently performed. Orientation to person, place, and time was intact. The Edinburgh Hand Preference Test yielded a score of +90, indicating strong right-hand dominance. Digit Span scores were four forward and three backward. On verbal memory assessment using the Buschke Selective Reminding Test, the patient learned five of 12 items after six learning trials, with a total learning score of 27/72. After approximately 20 min, free recall was 0/12, whereas 9/12 items were identified on recognition. On the WMS Visual Memory Subtest, the registration score was 6/14 and delayed retrieval was 2/14. Visuospatial perception and construction were relatively preserved. Language functions were generally preserved, although dysarthria and naming difficulty were noted. On the Stroop Test, completion time was 12 s for Card 1 and 50 s for Card 5, with four corrected errors. Categorical verbal fluency yielded 14 animal names, whereas lexical verbal fluency yielded a total of 20 words beginning with K, A, or S. The original clinical report additionally documented category-switching difficulty and planning difficulty; abstract thinking was scored as 3/6. Detailed available neuropsychological findings are summarized in Table 2.

Following this retrospective diagnostic formulation, oral B-complex vitamin supplementation was initiated at one tablet once daily. The prescribed regimen provided 250 mg/day of thiamine hydrochloride, 250 mg/day of pyridoxine hydrochloride, and 1 mg/day of cyanocobalamin. Based on the documented impairments in attention, memory, language-related performance, and executive functions, the patient was also enrolled in a structured cognitive rehabilitation program. The professional rehabilitation program lasted 10 weeks and consisted of one 60 min session per week, for a total of 10 sessions. Each session was delivered one-to-one by a neuropsychologist. The patient completed all 10 planned one-to-one 60 min sessions with the neuropsychologist.

Interventions included verbal and nonverbal attention and memory exercises; working-memory training; naming and speech exercises; and structured tasks targeting planning, abstract thinking, decision-making, categorization, inhibition, sequencing, and cognitive flexibility. Training was delivered using computer- and tablet-based programs, paper-and-pencil exercises, and other structured cognitive materials. A family-supported home program was additionally prescribed for daily practice and included reading, writing, arithmetic, oral-motor exercises, memory tasks, and activities designed to support information-processing speed.

Because depressive symptoms were identified clinically, supportive psychotherapy was also provided; however, depressive symptoms were not quantified using a standardized rating scale.

At the beginning of the rehabilitation period, family members reported that newly acquired information was generally retained for only approximately 1–2 h. During and after the 10-week rehabilitation period, they reported that the patient became able to retain and recall recent information and daily events for substantially longer periods, in some instances for approximately one day. They also reported greater independence in basic activities of daily living and recovery of some household abilities, including preparing simple meals.

These post-treatment changes were based primarily on clinical observation and family reports. The planned formal post-rehabilitation neuropsychological reassessment could not be completed because continuity of follow-up was not maintained. Consequently, the magnitude of cognitive change could not be objectively quantified, and the respective contributions of B-complex vitamin supplementation, improved nutritional status, spontaneous recovery, supportive care, and structured cognitive rehabilitation to the reported functional improvement cannot be distinguished in this single case.

## 3. Discussion

Wernicke encephalopathy (WE) is an acute neurological disorder resulting from thiamine deficiency and may lead to substantial neurological and cognitive sequelae when recognition and treatment are delayed. Although traditionally associated with chronic alcohol use, WE can occur in a wide range of non-alcohol-related settings, including gastrointestinal surgery, malignancy, prolonged vomiting, malabsorption, severe nutritional deprivation, and prolonged dependence on parenteral nutrition [1,2,3]. The present patient had never consumed alcohol, supporting a non-alcohol-related clinical context.

Thiamine stores are limited and may become depleted within a relatively short period when nutritional intake is severely compromised. Surgical stress, infection, malignancy, prolonged fasting, and increased carbohydrate metabolism may further increase thiamine requirements [1,3]. Thiamine is an essential cofactor for several enzymes involved in cerebral energy metabolism, including pyruvate dehydrogenase, alpha-ketoglutarate dehydrogenase, and transketolase. Deficiency may result in impaired ATP production, mitochondrial dysfunction, oxidative stress, excitotoxicity, and blood–brain barrier disruption [11,12]. Brain regions with high metabolic requirements, including the medial thalami, mammillary bodies, periaqueductal gray matter, brainstem oculomotor networks, and cerebellar structures, are particularly vulnerable [1,2].

The anatomical distribution of WE lesions accounts for many of its characteristic clinical manifestations. Involvement of brainstem and periaqueductal structures may produce nystagmus, diplopia, abducens palsy, or ophthalmoplegia, whereas cerebellar involvement may result in truncal instability and a wide-based gait. Damage involving the medial thalamic–mammillary circuitry may disrupt networks essential for memory formation and contribute to persistent anterograde amnesia and the subsequent Korsakoff syndrome [1,2,8,13].

The complete classic triad of altered mental status, oculomotor abnormalities, and ataxia is present in only a minority of patients with WE [2,4]. Diagnostic recognition may be particularly difficult in non-alcoholic postoperative patients because confusion, hallucinations, behavioral changes, or cognitive impairment can easily be attributed to postoperative delirium, infection, metabolic encephalopathy, medication effects, or systemic illness [1,5]. This diagnostic challenge was prominent in the present case. Confusion and visual hallucinations developed during a prolonged and complicated postoperative course and were initially interpreted within the context of delirium/metabolic encephalopathy. Oculomotor abnormalities, gait instability, and pronounced impairment in retention of recent information subsequently became evident, but WE was not specifically recognized during the acute phase.

Pancreaticoduodenectomy has previously been associated with WE, both in the early postoperative period and years after surgery [9,10]. In the present patient, severe restriction of oral and enteral intake, prolonged postoperative complications, infection and catabolic stress, and dependence on TPN created a setting of substantial nutritional vulnerability [3]. Importantly, however, the detailed composition of the TPN formulation could not be retrieved from the available medical documentation. It therefore remains unknown whether the patient received multivitamin or thiamine supplementation as part of TPN, whether supplementation was interrupted, or whether the amount administered was sufficient to meet metabolic requirements. Consequently, prolonged TPN should not be interpreted as an independently established cause of thiamine deficiency in this patient. Rather, it formed part of a broader postoperative nutritional-risk context characterized by severely restricted enteral intake and prolonged systemic illness.

The diagnosis of WE in this patient was reconstructed retrospectively rather than established during the acute postoperative period. Acute-phase thiamine measurements and other laboratory data specifically addressing thiamine status were unavailable. Nevertheless, the combination of prolonged nutritional compromise, altered mental status, marked recent-memory impairment, oculomotor abnormalities, gait ataxia, and characteristic anatomical abnormalities on the acute MRI provides substantial retrospective clinical support for WE. The subsequent persistence of severe anterograde memory impairment with executive dysfunction was considered compatible with a residual Korsakoff syndrome. This distinction is important because the available evidence supports a retrospectively reconstructed episode of WE followed by a chronic amnestic syndrome rather than a prospectively and biochemically confirmed acute diagnosis.

MRI findings in WE are supportive rather than diagnostic, and normal or subtle imaging findings do not exclude the disorder [14]. Typical abnormalities involve the medial thalami, mammillary bodies, periaqueductal gray matter, and other periventricular regions [6,14]. In our patient, the diagnostic hypothesis of previous WE arose primarily from the clinical history, neurological examination, and neuropsychological findings rather than from retrospective neuroimaging alone. The acute-phase MRI, which had not initially been interpreted as demonstrating findings suggestive of WE, was subsequently re-evaluated after the clinical diagnosis was considered. Bilateral medial thalamic and periaqueductal FLAIR hyperintensities were identified and were no longer evident on follow-up imaging approximately two years later.

The imaging findings were additionally assessed by an independent radiologist who was initially blinded to the clinical history and diagnostic hypothesis. The radiologist independently considered the acute-phase abnormalities to be pathological and confirmed their absence on follow-up MRI. After disclosure of the clinical information and the diagnostic suspicion of WE, the radiologist agreed that the anatomical distribution was compatible with WE. Although MRI remains supportive rather than independently diagnostic, this sequential independent assessment strengthens the radiological support for the retrospectively reconstructed clinical diagnosis.

Persistent cognitive impairment following WE is commonly dominated by anterograde amnesia, with variable retrograde memory impairment and executive dysfunction [8]. The neuropsychological profile in our patient was consistent with this pattern. Objective baseline testing showed Digit Span scores of four forward and three backward; limited verbal learning (5/12 items after six trials; total learning score of 27/72); no free recall after approximately 20 min (0/12) despite recognition of 9/12 items; WMS Visual Memory scores of 6/14 at registration and 2/14 at delayed retrieval; and an abstract-thinking score of 3/6, together with clinically documented category-switching and planning difficulties. These findings objectively document the presence and nature of the cognitive impairment before rehabilitation; however, they should not be interpreted as objective evidence of subsequent treatment-related cognitive change because formal post-treatment testing could not be completed.

Rehabilitation studies in Korsakoff syndrome suggest that some patients may retain the capacity for functional learning and may benefit from structured practice, procedural learning, external memory support, and error-reduction approaches [15,16,17]. These findings provide a rationale for rehabilitation even when persistent amnesia is present. In the present patient, the rehabilitation program consisted of 10 weekly 60 min professional sessions targeting attention, verbal and nonverbal memory, working memory, language-related tasks, planning, abstract thinking, inhibition, sequencing, decision-making, categorization, and cognitive flexibility. A daily family-supported home program was also prescribed. The intervention did not constitute a predefined errorless-learning or procedural-learning protocol, and these specific approaches should therefore not be attributed to the treatment delivered in this case. The sessions were delivered individually by a neuropsychologist.

The case described by Palmirotta et al. [18] provides a useful comparison. Their patient had non-alcoholic Wernicke–Korsakoff syndrome in the setting of severe pancreatic disease and underwent intensive rehabilitation combined with high-dose thiamine treatment, with cognitive outcome documented through formal neuropsychological reassessment. In contrast, our patient developed the syndrome following a complicated pancreaticoduodenectomy with prolonged nutritional compromise, the syndrome was recognized retrospectively approximately two years later, and the patient underwent a less intensive outpatient cognitive rehabilitation program. Oral B-complex vitamin supplementation was initiated after the retrospective diagnostic formulation at one tablet once daily, providing 250 mg/day of thiamine hydrochloride (together with 250 mg/day of pyridoxine hydrochloride and 1 mg/day of cyanocobalamin). Most importantly, post-rehabilitation outcome in our case was based primarily on clinical observation and family reports rather than repeat standardized neuropsychological testing. These differences preclude direct comparison of treatment efficacy and help define both the strengths and limitations of the present case [18].

During follow-up, family members reported that the patient’s ability to retain and recall recently acquired information improved from approximately 1–2 h to substantially longer periods, sometimes approaching one day, and that she became more independent in basic activities of daily living and regained some household abilities. These observations are clinically relevant because they describe change in everyday function; however, they are informant-reported outcomes and are subject to recall and observer bias. Moreover, B-complex vitamin supplementation, improved nutritional intake, supportive psychotherapy, spontaneous neurological recovery, and cognitive rehabilitation occurred within the broader follow-up period. Their individual contributions to the observed functional change cannot be separated in a single uncontrolled case.

Depressive symptoms represent an additional potential confounding factor. The patient received supportive psychotherapy because depressive symptoms were identified clinically, but these symptoms were not quantified using a standardized instrument such as the Beck Depression Inventory or Hamilton Depression Rating Scale. Because mood symptoms may influence attention, memory, processing speed, motivation, and executive performance, their contribution to both baseline cognitive performance and subsequent functional change cannot be determined.

The principal limitations of this report include its retrospective diagnostic formulation, absence of acute-phase biochemical confirmation of thiamine deficiency, inability to verify the thiamine or multivitamin content of the TPN regimen, absence of standardized quantification of depressive symptoms, reliance on family reports for post-rehabilitation functional change, and the lack of a formal post-treatment neuropsychological reassessment. Although the imaging findings were additionally reviewed by an independent radiologist, their relationship to WE was necessarily interpreted within the later clinical context. Furthermore, because B-complex vitamin supplementation, nutritional recovery, supportive care, and rehabilitation were not isolated interventions, no causal inference regarding the efficacy of cognitive rehabilitation can be made.

Despite these limitations, the principal contribution of this case lies in its longitudinal characterization of an underrecognized postoperative presentation of probable Wernicke encephalopathy following pancreaticoduodenectomy, with integration of retrospective acute-phase neuroimaging, detailed baseline neuropsychological findings, a persistent clinical syndrome compatible with Korsakoff syndrome, and subsequent rehabilitation follow-up. The case underscores the importance of considering WE early in nutritionally vulnerable postoperative patients and illustrates both the potential value and the methodological limitations of documenting later functional change when standardized follow-up testing is unavailable.

## 4. Conclusions

This case highlights the diagnostic challenge of Wernicke encephalopathy in a non-alcoholic patient with a complicated postoperative course following pancreaticoduodenectomy. In patients with prolonged restriction of oral or enteral intake who develop otherwise unexplained confusion, memory impairment, oculomotor abnormalities, or gait ataxia, WE should be considered even when postoperative delirium or metabolic encephalopathy initially appears more likely.

The present case also illustrates the importance of integrating the clinical history with retrospective neuroimaging when the diagnosis was not recognized during the acute phase. In our patient, the diagnosis was first suspected on the basis of the later clinical and neuropsychological findings; subsequent re-evaluation of the acute-phase MRI, together with radiological review, identified bilateral medial thalamic and periaqueductal abnormalities compatible with WE, whereas these abnormalities were no longer evident on follow-up imaging. An additional independent radiologist, initially blinded to the clinical history and diagnostic hypothesis, confirmed that the acute-phase abnormalities were pathological and absent on follow-up imaging. The persistent amnestic and executive deficits were consistent with a residual Korsakoff syndrome.

During follow-up after initiation of B-complex vitamin supplementation, nutritional optimization, and structured cognitive rehabilitation, the patient’s family reported improvement in retention of recent information, daily functioning, and independence. However, because a formal post-treatment neuropsychological reassessment was not completed and several interventions occurred during the same period, these functional changes cannot be attributed specifically to cognitive rehabilitation.

Greater awareness of WE in nutritionally vulnerable postoperative patients may facilitate earlier recognition and treatment before persistent cognitive sequelae develop. Future prospective studies should further define the incidence and risk factors for thiamine deficiency after major gastrointestinal surgery, evaluate optimal strategies for thiamine monitoring and prophylactic supplementation, and determine the role and most effective components of structured cognitive rehabilitation in patients with persistent Wernicke–Korsakoff-related cognitive impairment.

## Figures and Tables

**Table 1:** Clinical timeline of the case.

Time Point	Clinical Events and Findings	Management/Outcome
Month 0	Whipple procedure for pancreatic head adenocarcinoma; the first two postoperative weeks were uneventful.	Surgical treatment completed.
From postoperative week 2 through the prolonged postoperative course	Fever led to detection of an anastomotic leak. The patient was managed for approximately four months with an open abdomen and intra-abdominal vacuum system. Oral intake was severely restricted, and nutrition was primarily maintained with TPN.	Prolonged postoperative management with limited enteral intake and long-term TPN. Detailed records of the TPN formulation were unavailable; therefore, concomitant multivitamin or thiamine supplementation could not be retrospectively verified.
Approximately 1.5 months postoperatively	Fluctuating confusion, visual hallucinations, marked impairment in retention of recent information, dizziness, diplopia, jerky eye movements, and severe gait unsteadiness developed.	Neurological and psychiatric evaluations and cranial imaging were performed. In the context of the complicated postoperative course, the presentation was initially interpreted as postoperative delirium/metabolic encephalopathy, and symptomatic treatment was recommended.
Approximately 5.5 months after surgery and early post-discharge period	General medical condition improved sufficiently for discharge. Visual misperceptions gradually diminished, and dizziness and diplopia substantially improved; however, marked recent-memory impairment and balance difficulties persisted.	Oral nutritional intake was progressively increased with family support, with particular attention to a protein- and vitamin-rich diet. No additional medically prescribed nutritional supplementation was reported during this period.
Approximately 2 years after surgery	Neurological reassessment demonstrated persistent anterograde amnesia, bilateral gaze-evoked nystagmus, cerebellar dysarthria, tremor, and ataxic gait. Review of the acute-phase MRI demonstrated bilateral medial thalamic and periaqueductal FLAIR hyperintensities, whereas these abnormalities were no longer evident on follow-up MRI. Neuropsychological assessment documented deficits in attention, verbal and nonverbal memory, and executive functions.	The acute neurological syndrome was retrospectively considered compatible with WE, and the persistent amnestic syndrome was considered compatible with residual Korsakoff syndrome. Oral B-complex vitamin supplementation was initiated at one tablet daily (250 mg/day thiamine hydrochloride, 250 mg/day pyridoxine hydrochloride, and 1 mg/day cyanocobalamin).
Subsequent 10-week rehabilitation period	The patient participated in a structured cognitive rehabilitation program delivered one-to-one by a neuropsychologist, consisting of one 60 min session per week for 10 weeks, together with a daily family-supported home program. Supportive psychotherapy was also provided for clinically identified depressive symptoms.	Rehabilitation targeted attention, verbal and nonverbal memory, working memory, naming and speech, planning, abstract thinking, decision-making, categorization, inhibition, sequencing, and cognitive flexibility.
During and after rehabilitation follow-up	Family members reported longer retention of recently acquired information and daily events, with improvement from approximately 1–2 h at baseline to substantially longer periods, in some instances approximately one day. They also reported greater independence in basic activities of daily living and recovery of some household activities, including preparation of simple meals.	A planned formal post-rehabilitation neuropsychological reassessment could not be completed because continuity of follow-up was not maintained. Therefore, the reported changes remained informant-reported and could not be objectively quantified or attributed specifically to rehabilitation.

**Table 2:** Baseline neuropsychological findings before cognitive rehabilitation.

Test/Measure	Cognitive Domain	Available Result	Clinical Interpretation
Digit Span—Forward	Attention/immediate auditory span	4	Forward span = 4; the original clinical report described attention as below expected limits.
Digit Span—Backward	Attention/working memory	3	Backward span = 3; the original clinical report described attention/working memory as below expected limits.
Buschke Selective Reminding Test	Verbal learning	5/12 items learned after six trials	Five of 12 items were acquired after six learning trials.
Buschke Selective Reminding Test	Total verbal learning	27/72	Total learning score = 27/72.
Delayed Free Recall (~20 min)	Delayed verbal recall	0/12	No items were freely recalled after approximately 20 min.
Recognition (~20 min)	Verbal recognition	9/12	Nine of 12 items were recognized; recognition exceeded free recall.
WMS Visual Memory Subtest	Nonverbal memory—registration	6/14	Registration score = 6/14.
WMS Visual Memory Subtest	Nonverbal memory—delayed retrieval	2/14	Delayed retrieval score = 2/14.
Cube and Drawing Tasks	Visuospatial perception and construction	4/5	Score = 4/5; visuospatial/construction performance was relatively preserved.
Boston Naming Test	Naming	22/27	Naming score = 22/27; language functions were otherwise generally preserved.
Abstract Thinking	Executive/conceptual function	3/6	Abstract-thinking score = 3/6.
Judgment	Executive function	6/6	Judgment score = 6/6.
Calculation	Calculation	6/8	Calculation score = 6/8.
Stroop Test	Inhibitory control/mental control	Card 1: 12 s; Card 5: 50 s; 4 corrected errors	Card 1 was completed in 12 s and Card 5 in 50 s, with four corrected errors; category-switching difficulty was noted clinically.
Categorical and Lexical Verbal Fluency	Executive function/verbal fluency	Categorical: 14 animal names; lexical (K-A-S): 20 words total	Categorical fluency = 14 animal names; lexical fluency (K-A-S) = 20 words in total.
Planning Tasks	Executive function	Numerical score unavailable	Planning difficulty was documented qualitatively in the original clinical report; no numerical score was retained.
Edinburgh Hand Preference Test	Hand preference	+90	Score = +90, indicating strong right-hand dominance.
Clock Drawing Test	Executive/visuospatial function	Numerical score not available	No separate quantitative result was documented in the retained clinical report.

Note: Qualitative interpretations included in the original version of this table were taken from the original neuropsychological clinical report and were not assigned retrospectively by the authors. In response to the reviewer, the table has been revised to prioritize the available numerical and descriptive findings. Where corrected or standardized scores, percentile values, detailed test-version information, or reliable normative reference values were not available in the retained documentation, no additional retrospective severity classification was assigned.

## Data Availability

The data presented in this case report are contained within the article. Further inquiries may be directed to the corresponding author.

## Abbreviations

WE, Wernicke encephalopathy; TPN, total parenteral nutrition; MRI, magnetic resonance imaging; FLAIR, fluid-attenuated inversion recovery.
